# miR-155 induced transcriptome changes in the MCF-7 breast cancer cell line leads to enhanced mitogen activated protein kinase signaling

**DOI:** 10.18632/genesandcancer.33

**Published:** 2014-09

**Authors:** Elizabeth C. Martin, Adrienne E. Krebs, Hope E. Burks, Steven Elliott, Melody Baddoo, Bridgette M. Collins-Burow, Erik K. Flemington, Matthew E. Burow

**Affiliations:** ^1^ Department of Medicine-Section of Hematology and Medical Oncology, Tulane University, New Orleans, LA; ^2^ Tulane Cancer Center, Tulane University, New Orleans, LA; ^3^ Department of Pathology, Tulane University, New Orleans, LA; ^4^ Department of Pharmacology, Tulane University, New Orleans, LA

**Keywords:** microRNA-155, breast cancer, MAPK, p38, 3′UTR, RNA-seq

## Abstract

A single microRNA (miRNA) has the potential to regulate thousands of genes and thus govern multiple signaling pathways at once. miR-155 is an oncogenic miRNA which regulates many cellular pathways, designating it as a multifaceted regulator of proliferation, chemo-resistance, and apoptosis. While many singular targeted effects of miR-155 have been defined and an oncogenic role has been attributed to miR-155 expression, the global effect of miR-155 on the cellular transcriptomes of an ER^+^ breast cancer cell line has yet to be determined. Here we demonstrate that miR-155 expression increases tumorigenesis *in vivo* and we determine miR-155 mediated transcriptome changes through next generation sequencing analysis. miR-155 expression alters many signaling pathways, with the chief altered pathway being the MAPK signaling cascade and miR-155 induces shortening of target mRNA 3′UTRs and alternative isoform expression of MAPK related genes. In addition there is an observed increase in protein phosphorylation of components of MAPK signaling including ERK1/2 and AP-1 complex members (Fra-1 and c-Fos) as well as elevated gene expression of MAPK regulated genes Zeb1, Snail, Plaur, and SerpinE1.

## INTRODUCTION

microRNAs (miRNAs) are small non-coding RNAs known to mediate cancer progression [1-4]. Among cancers of the breast, expression profiling of miRNAs shows correlations between miRNA expression and receptor status for estrogen receptor (ER), progesterone receptor (PgR), and V-Erb-B2 avian erythroblastic leukemia viral oncogene homolog 2 (HER2/Nue) [5, 6]. In addition to a prognostic correlation, miRNA expression correlates with all facets of breast cancer progression including proliferation, endocrine resistance, and metastasis [7]. One miRNA, microRNA-155 (miR-155) is known to regulate multiple aspects of breast cancer progression in addition to demonstrating a correlation with loss of receptor status [2, 3, 8, 9]. Located within the noncoding B cell integration cluster (BIC) gene, microRNA-155 (miR-155) was first discovered through retroviral integrations in B cell lymphomas [12]. Currently miR-155 is found to be amplified in many cancers, irrespective of retroviral integration [13]. miR-155 expression is such a powerful mutagen that transgenic mice over-expressing this single miRNA in B-cells demonstrate an accumulation of pre-B cells and eventually develop B-cell malignancies lymphoma/leukemia [14]. As one of the more highly evaluated miRNAs, many targets are known and miR-155 is an established oncogenic miRNA [2, 3, 15]. Through targeting of genes such as SOCS1, TP53BP1, and FOXO3a miR-155 is able to enhance cellular proliferation and survival of breast cancer cells [2, 10, 11].

miR-155 is known to regulate cellular proliferation and survival through multiple mechanisms, including the mitogen activated protein kinases (MAPKs) signaling pathways. miR-155 is a positive multi-faceted regulator of MAPK signaling, intervening at heterogeneous points along the signaling cascade to enhance signaling. Through targeting of inositol polyphosphate-5-phosphatase (SHIP1) miR-155 enhances extracellular signal-regulated kinases (ERK) activation [17]. In a glioblastoma cell line, miR-155 expression enhanced ERK phosphorylation and cellular proliferation while alternatively, the loss of miR-155 expression led to decreased ERK activation in B-cells [18, 19]. Downstream MAPK effectors such as AP-1 complex member c-Jun, are enhanced through miR-155 targeting of DET1. Targeting of DET1 stabilizes c-Jun transcripts and subsequently increases AP-1 activity [20].

Here we demonstrate through next generation sequencing analysis, global changes in both MAPK ERK1/2 and p38 gene expression due to overexpression of miR-155. In addition, miR-155 enhanced MAPK signaling which was observed through increased MAPK and effector protein phosphorylation.

## RESULTS

### Over-Expression of miR-155 Enhances Tumorigenesis *in vivo*

**Figure 1:**
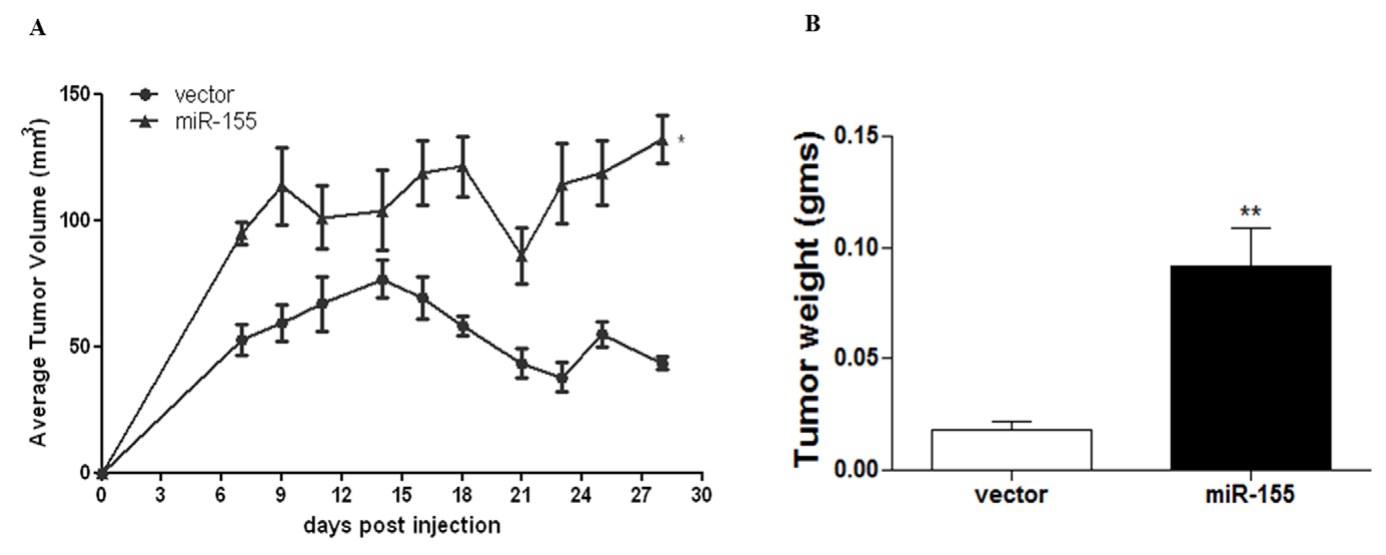
miR-155 Expression Enhances Tumorigenesis *in vivo* ovariectomized SCID/CB-17 female mice injected bilaterally with 5×10^6^ MCF-7-vector cells or MCF-7-miR-155 cells, n=5 animals/group (A) Tumor volume, points represent average tumor volume ± SEM starting at day 7 post injection, measurements were carried out until necroscopy (day 28). (B) final tumor weight at necroscopy (day 28). * significantly different p < 0.05, ** significantly different p < 0.01

miR-155 has previously been defined as an oncogenic miRNA enhancing proliferation and survival [2, 3, 40, 41]. To observe a comprehensive effect of miR-155 over-expression on ER^+^ breast cancer tumorigenesis, MCF-7 cells over-expressing miR-155 (MCF-7-miR-155) were generated and validated as previously described [52]. Cells were then inoculated in ovariectomized CB-17/SCID female mice in the mammary fat pad (MFP). At necropsy (day 28 post cell injection), final tumor volume and weight was greater for MCF-7-miR-155 tumors compared to vector tumors (Figure 1A and 1B respectively), demonstrating that miR-155 expression enhances basal tumorigenesis *in vivo*.

### 3′UTR Shortening Leads to Increased Expression of miR-155 MAPK Target Genes

**Figure 2:**
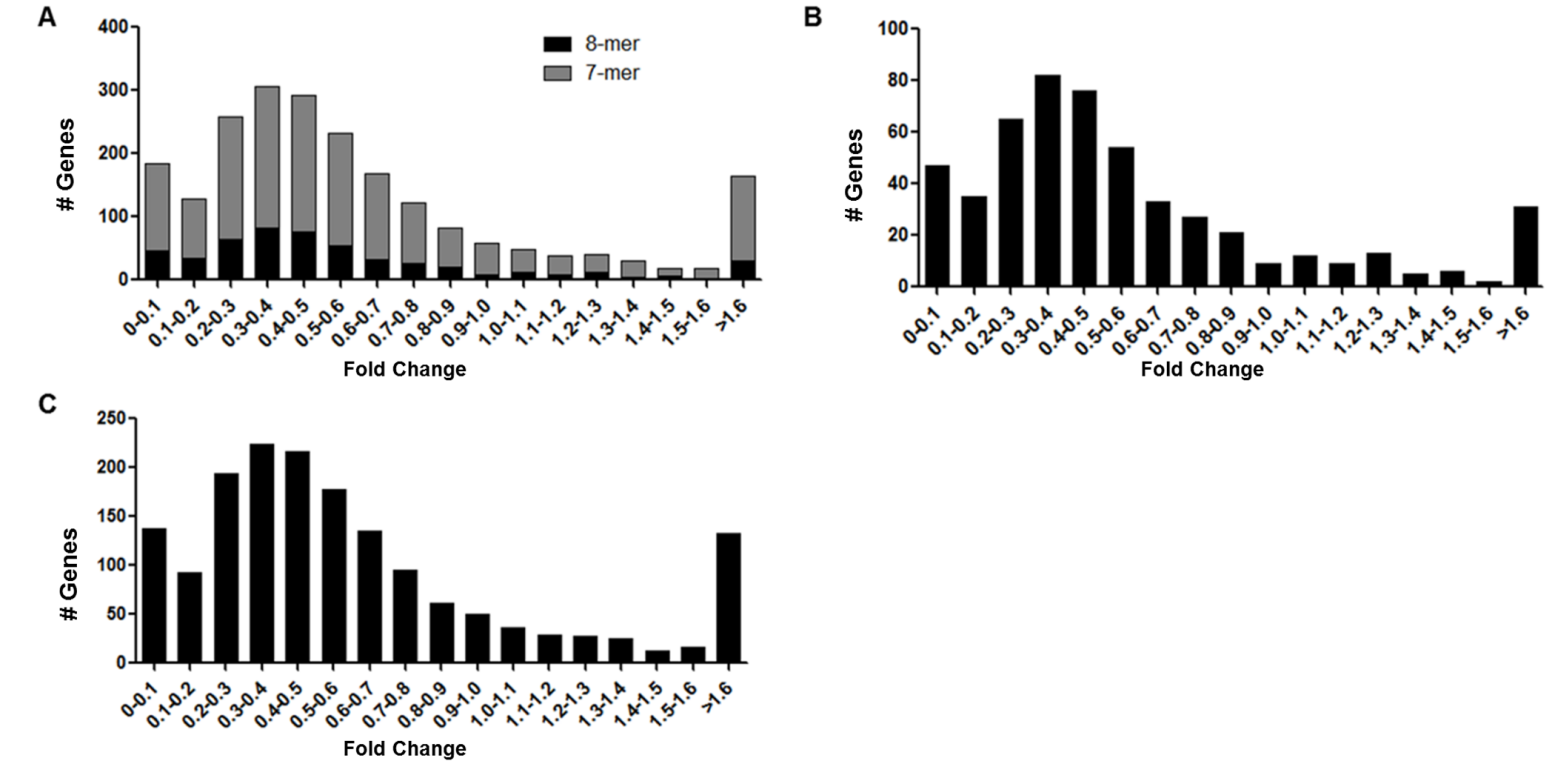
Next Generation Sequencing Analysis of miR-155 in the MCF-7-miR-155 Cell Line MCF-7-vector and –miR-155 cell lines were extracted for total RNA and were analyzed through next generation sequencing Genes containing any miR-155 seed site were pooled and further analyzed. Results represent fold change of predicted miR-155 targets with (A) 8mer or 7mer site (B) 8mer site (C) 7mer site. X-axis depicts fold change in change expression between MCF-7-miR-155 cell line and vector, y-axis demonstrates number of genes.

**Figure 3:**
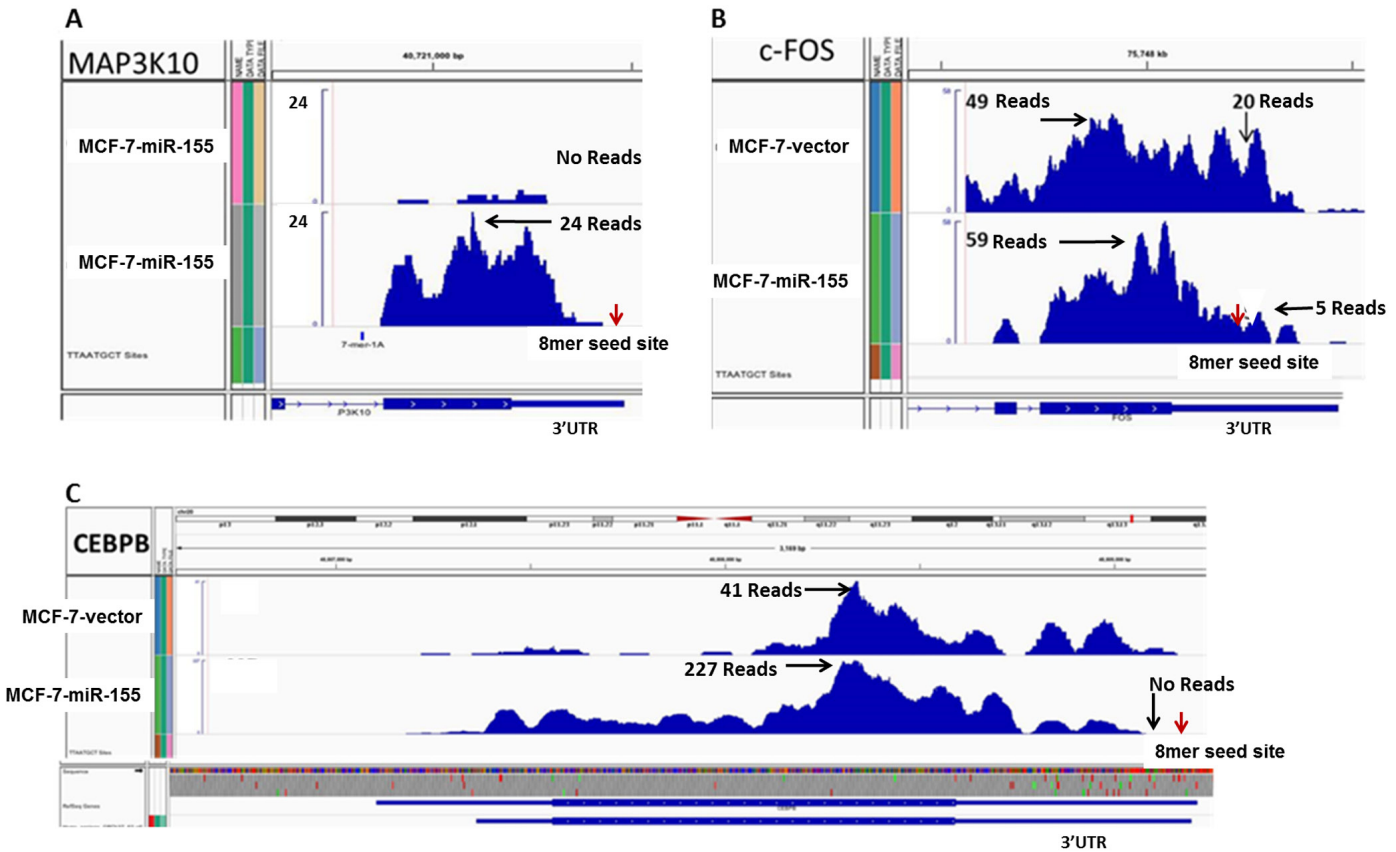
Genes Containing miR-155 Seed Site Demonstrate Loss of 3′UTR in the MCF-7-miR-155 Cell Line Next generation sequencing analysis, representative pictures of 3′UTR of MAPK associated genes with miR-155 seed site (A) MAP3K10 (B) c-FOS and (C) CEBPβ. Results represent raw reads and reference genome is HG19

To determine an underlying mechanism for the observed miR-155 induced tumorigenesis *in vivo*, MCF-7-miR-155 and –vector cell lines were analyzed with next generation sequencing. Subsequently, all miR-155 seed sites, identified through our in-house Seedfinder program, were analyzed. Interestingly, while many predicted miR-155 targets demonstrated an overall repression of expression, having fold expression levels of 0.5 or lower compared to the vector cell line, some predicted targets of miR-155 demonstrated an increase in expression levels (fold >1.5) (Figure 2A). This increase in expression was observed in miR-155 predicted targets irrespective of whether or not the seed site was an 8-mer or 7-mer site (Figure 2B and 2C respectively). To determine the possible cause for increased expression of mRNAs containing a miR-155 predicted target sites we examined all miR-155 predicted targets which contained an 8-mer-seed site and had an increase in expression over 1.5 fold. Of the thirty targets showing increased expression, fourteen of these targets demonstrated very low reads (below 10) in both the MCF-7-vector and –miR-155 cell line and were therefore not chosen for further analysis. Increased cellular proliferation as well as exogenous stimulation can be attributed to shortening of 3′UTR [42, 43]. This has previously been demonstrated in miR-155 overexpressing cell lines, where miR-155 target site was lost due to 3′UTR shorting [44]. As our miR-155 cell line showed increased tumorigenesis, we next sought to explore the possibility that variability of 3′UTR and loss of miR-155 target sites occurred in our miR-155 cell line. Of the remaining sixteen targets, ten transcripts with isoforms demonstrating sufficient levels of expression, showed a loss of 3′UTR expression at the miR-155 seed site in the MCF-7-miR-155 cell line compared to –vector (Table 1). One miR-155 target, MAP3K10 demonstrated loss of 3′UTR in both the vector and miR-155 cell lines (Figure 3A).

**Table 1:** Loss of 3′UTR in Genes with miR-155 8-mer Seed Sites

Gene	MCF-7-miR-155 3′UTR Shortening	Relative Expression miR-155/vector
SPIN2B	Yes	1.516575
GLT25D1	No	1.557417
MAP3K14	Yes	1.849516
CARS2	Yes	1.862035
ARVCF	Yes	2.292287
AGTRAP	Yes	2.816578
MYLK	No	3.222538
CARD10	Yes	3.237354
IRF2BP2	Yes	3.330693
CSNK1G2	No	3.472894
TRMT61A	Yes	3.627442
MAP3K10	Yes	3.813621
CEBPB	Yes	4.871474
PRKAR1B	Yes	7.552545
SOCS1	No	8.080313
SPOCK1	No	20.33727

**Figure 4:**
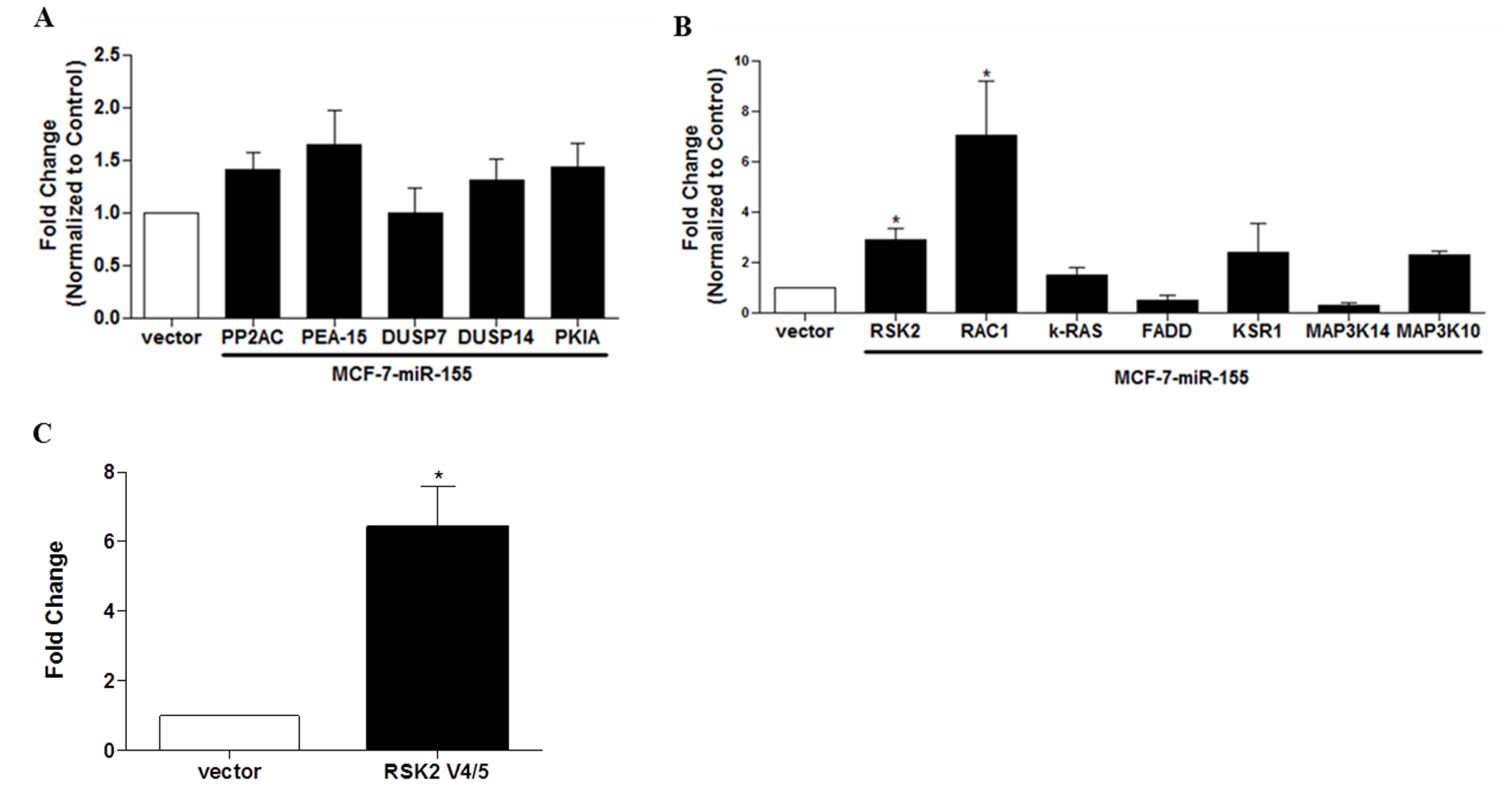
Expression of MAPK Associated Genes with miR-155 Seed Sites Following miR-155 Over-Expression in MCF-7 Cells MCF-7-vector and MCF-7-miR-155 cells were harvested for qPCR for genes which contain a miR-155 seed site. Genes were selected that (A) repress MAPK signaling (PP2AC, PEA-15, DUSP7, DUSP14, PKIA) and (B) are associated with activated MAPK signaling (RSK2, RAC1, k-RAS, FADD, KSR1, MAP3K14, MAP3K10) (C) qPCR for MCF-7-miR-155 cell line expression of RSK2 isoform variant 4 and 5. Normalization was to actin and MCF-7-vector cells designated as 1. Error bars represent SEM. * p < 0.05. n = 3

Due to the fluid nature of miR-155 over-expression and mRNA target selection, we next used the online pathway interaction database (PID) to determine the top pathways predicted to be targeted by miR-155 through BioCarta derived analysis [http://www.cancer.gov]. Genes which contain a miR-155 seedsite were loaded into the pathway data base to determine if a correlation existed between genes containing a miR-155 seed site and a specific pathway. Relevant pathways were determined based on the number of associated proteins and the relative affinity each protein had for a particular pathway and a p value was assigned to each pathway to demonstrate significance of correlation, the top pathway contained the greatest significant of correlation. The results from this analysis showed that many of the top pathways predicted to be targeted by miR-155 were pathways regulated by growth factors and the cell cycle (Table 2). In addition to miR-155 predicted targets we analyzed all genes that were suppressed by miR-155 over-expression and all genes demonstrating an increase in expression (Table 2). Interestingly, pathway predictions based on down-regulated genes corresponded to MAPK signaling and cell death. Of genes demonstrating increased levels of expression, many were associated with proliferation with the top pathway being MAPK signaling and subsequent MAPK related pathways (p38 MAPK signaling, ERK1/2 MAPK signaling pathway, RAS signaling pathway). As miR-155 has predicted targets of MAPK signaling which would both activate and repress the pathway, we next sought to validate MAPK associated miR-155 predicted targets. Both activators (RSK2, k-RAS, KSR1, FADD, and RAC1) and repressors (PP2AC, DUSP7, DUSP14, and PEA-15) were chosen for further analysis with qPCR. Many miR-155 MAPK associated predicted targets which are repressors of MAPK signaling showed no significant change in either direction (Figure 4A) while activators of MAPK pathway were either unchanged or demonstrated significant increases in expression levels (Figure 4B). To determine a mechanism for the changes observed in MAPK targets which would enhance MAPK signaling, the 3′UTRs of MAPK activators RAC1, RSK2, k-RAS, and KSR1 were investigated. Only RAC1 and MAP3K10 demonstrate 3′UTR shortening and loss of miR-155 targeting (Table 3). RSK2 was the only predicted miR-155 target demonstrating a significant increase in expression without a subsequent loss of 3′UTR; we sought to further evaluate the mechanisms responsible for the increase in RSK2 expression. Isoform variance is known to exist among gene transcripts and is known to result in loss of miRNA targeting through altered isoform expression [44]. qPCR was performed for the RKS2 isoform variants 4 and 5 which demonstrate a truncated transcript and do not contain the same 3′UTR as the other RSK2 transcripts. A significant increase in expression of isoforms 4/5 of RSK2 is observed in the miR-155 over-expressing cell line (Figure 4C). Together, these data indicate that mediators of MAPK signaling are increased in the MCF-7-miR-155 cell line

**Table 2:** Analysis of miR-155 Regulated Pathways

Up Regulated Pathways	Down Regulated Pathways	miR-155 Targeted Pathways
**MAPK Signaling Pathway**	Rho cell motility signaling pathway	Rac1 cell motility signaling pathway
RHO cell motility signaling pathway	Rac1 cell motility signaling pathway	Regulation of bad phosphorylation
Integrin signaling pathway	Ras signaling pathway	Bioactive peptide induced signaling pathway
Rac1 Cell Motility Signaling Pathway	Integrin signaling pathway	Transcription factor creb and its extracellular signals
Inhibition of Cellular Proliferation by Gleevec	Role of Brca1 Brca2 and Atr in cancer susceptibility	Growth hormone signaling pathway
ERK and PI3K collagen in corneal epithelia	Proteasome complex	Rho cell motility signaling pathway
Negative effectors of Fas and TNF	Hiv-1 nef: negative effector of fas and tnf	Cell cycle: g1/s check point
Bioactive peptide induced signaling pathway	Cyclin E destruction pathway	Co-stimulatory signal during t-cell activation
**P38 MAPK signaling pathway**	Ifn alpha signaling pathway	Igf-1 signaling pathway
Induction of apoptosis through DR3 and DR4/5 death receptors	**Map kinase signaling pathway**	**Ras signaling pathway**
Nuclear receptors coordinate the activities of chromatin remodeling complexes and coactivators to facilitate initiation of transcription in carcinoma cells	ERK and PI3 kinase are necessary for collagen binding in corneal epithelia	Phospholipase c signaling pathway
Ras signaling pathway	Hypoxia and p53 in the cardiovascular system ATM	Hiv-1 nef: negative effector of fas and tnf
Control of skeletal myogenesis by HDAC and calcium/calmodulin dependent kinase (CAMK)	Caspase cascade in apoptosis	Human cytomegalovirus and map kinase pathways
Growth hormone signaling pathway	Rb tumor suppressor/checkpoint signaling in response to dna damage	Akt signaling pathway
**ERK1/ERK2 MAPK signaling pathway**	D4GDI signaling pathway	Egf signaling pathway
IL-7 signal transduction	Cell cycle: g1/s check point ATM	Multiple antiapoptotic pathways from igf-1r signaling lead to bad phosphorylation
FC epsilon receptor I signaling in mast cells	Links between pyk2 and map kinases	Trka receptor signaling pathway
IL-22 soluble receptor signaling pathway	Transcription factor creb and its extracellular signals	**Map kinase signaling pathway**
Apoptotic DNA-fragmentation and tissue homeostasis	CDC25 and chk1 regulatory pathway in response to dna damage	Role of pi3k subunit p85 in regulation of actin organization and cell

**Table 3:** Genes with miR-155 Seed site and of Loss of 3′UTR

Gene	7mer sites	8mer sites	3′UTR shortening
RSK2	1	0	No
Rac1	1	0	Yes
k-Ras	3	0	No
KSR1	0	1	No
MAP3K14	0	1	No
MAP3K10	0	1	Yes

### miR-155 Expression Enhances MAPK Signaling in MCF-7 Breast Cancer Cell Line

**Figure 5:**
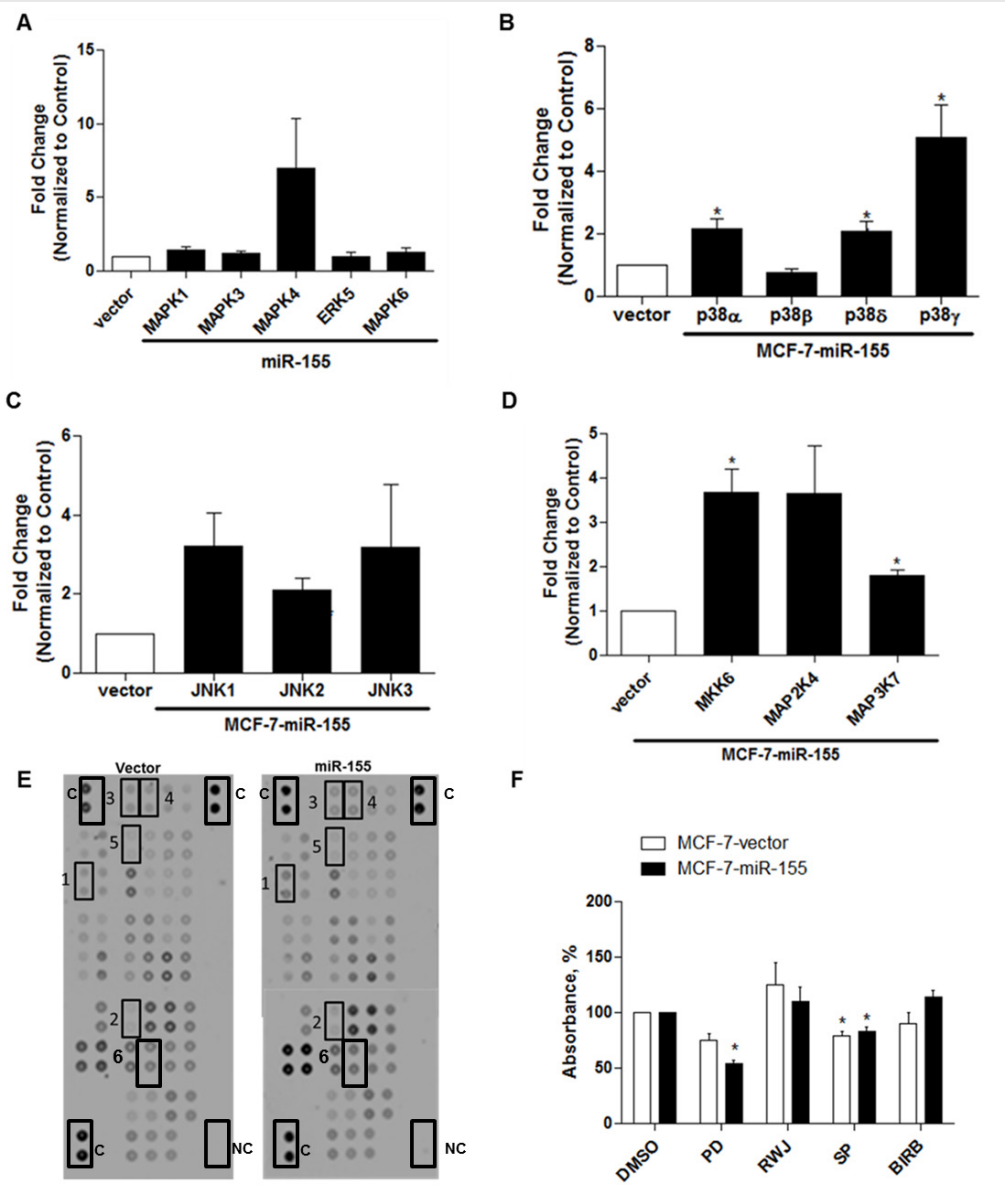
miR-155 Alters MAPK Signaling

To better assess the global effect of miR-155 on MAPK signaling, all MAPK genes demonstrating detectable expression levels were pooled from next generation sequencing data and analyzed. MAPK signaling is multifaceted, including four major signaling families, the ERKs, JNKs, p38s, and ERK5 [24]. Following analysis of sequencing data, many isoforms of p38 were up-regulated (MAPK11, MAPK12, MAPK13) while the p38α (MAPK14) isoform was repressed. To validate observed changes in sequencing, qPCR was performed to confirm changes in MAPK signaling following enhanced miR-155 expression. Expression levels of all MAPKs (MAPK1-MAPK14) and some associated MAPKKKs (MAP3K) and MAPKKs (MAP2K7, MAP2K4, MKK4) were quantified. qPCR results demonstrate no significant change in MAPKs 1-7 (Figure 5A). A significant overall increase in expression levels of p38 isoforms (α, δ, and γ) and p38 associated MAPKKS (MKK6 and MAP2K4) was observed (Figure 5B and 5D). The JNK pathway showed no significant change (Figure 5C). To determine overall changes in protein phosphorylation a protein kinase array was performed comparing the MCF-7-vector and MCF-7-miR-155 cell lines. Array results demonstrated ERK1/2 to have increased phosphorylation in the MCF-7-miR-155 cell line (Figure 5E). These data suggest that miR-155 overexpression affects MAPK signaling through alterations in the p38 and ERK1/2 signaling cascades. To better determine which MAPK pathway is involved in increasing cellular proliferation, crystal violet assay was performed on MCF-7-miR-155 cells and vector following treatment of MAPK inhibitors for 72 hours. Inhibition of MEK1/2 signaling led to significant repression of cellular proliferation in the MAPK-miR-155 cell line but not the MCF-7-vector cell line, this suggest that MAPK/ERK1/2 signaling is involved in miR-155 driven proliferation (Figure 5F).

MCF-7-vector and –miR-155 cell lines were harvested for qPCR to determine basal expression levels for (A) MAPKs 1-6, (B) p38 isoforms (C) JNK1-3 and (D) MAPK associated genes. Normalization was to actin and vector cells designated as one. Error bars represent SEM, n > 3. (E) Human Phospho-Kinase Protein Array for miR-155 Targets. Representative membranes from 2 independent experiments evaluating a broad range of phosphorylation targets for MCF-7 Vector and miR-155 cell lines. Selected targets are ERK1/2 T202 Y204 (1), p70 S6 Kinase T389 (2), Src Y419 (3), mTOR S2448 (4), CREB S133 (5), and RSK1/2/3 S380/ S386/S377 (6). NC= negative control, C= reference spot. (F) Crystal violet assay for cell survival following treatment with MAPK pathway specific inhibitors PD184352 (MEK1/2 inhibitor), SP600125 (broad spectrum JNK inhibitor), RWJ67657 (p38α and p38β inhibitor, does not inhibit p38γ or δ), BIRB (broad spectrum p38 inhibitor). n = 3. * significantly different p < 0.05.

### AP-1 Signaling is altered in MCF-7 Breast Cancer Cells Over-Expressing miR-155

**Figure 6:**
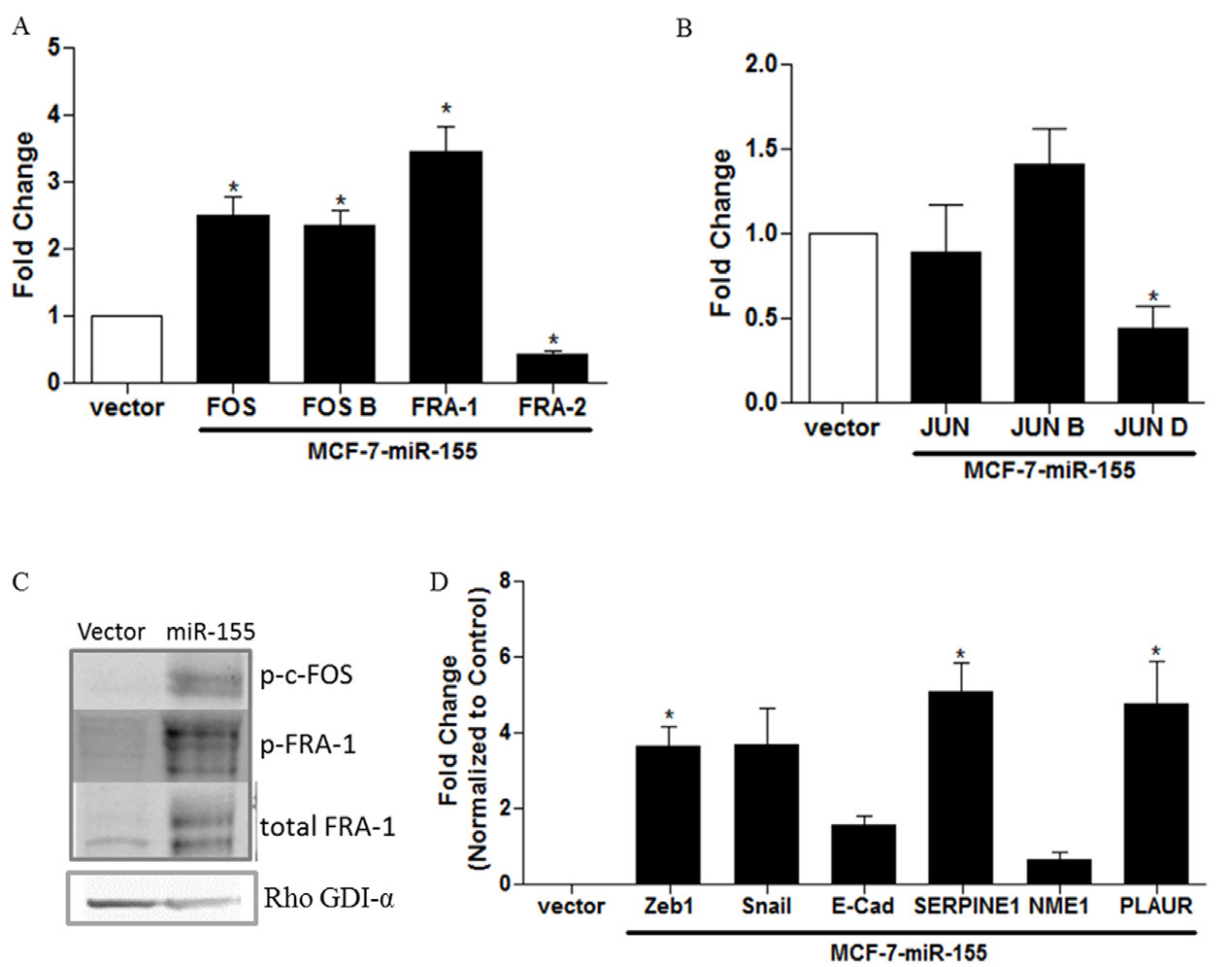
miR-155 Induces Changes in AP-1 Signaling. MCF-7-vector and —miR-155 cells were collected for RNA extraction and qPCR was performed to determine basal expression levels of AP-1 complex components for (A) FOS family members and (B) Jun family members, n = 3 Normalization was to actin and —vector cells designated as 1 (C) Western blot analysis in MCF-7-miR-155 and —vector cells for p-c-FOS, p-c-FRA-1, total FRA-1. Normalization was to RHO GDI-α, n = 3. (D) qPCR was performed to determine basal expression levels of downstream MAPK regulated genes ZEB1, SNAIL, E-cadherin, SERPINE1, NME1, and PLAUR in MCF-7-vector cells versus —miR-155. Normalization was to actin and vector cells designated as 1. Error bars represent SEM, * p < 0.05.

MAPK signaling ultimately results in activation of transcription factors such as the AP-1 complex and initiates gene transcription. The p38 isoforms α, δ, and γ demonstrated significant increases in expression in the miR-155 overexpressing cell lines (Figure 5B) as did the MAPK activator RAC1 and the downstream signaling molecule RSK2 (Figure 4B), all of which are known to enhance AP-1 signaling. We next sought to evaluate AP-1 complex members to determine basal level differences in Fos family members (c-Fos, FosB, Fra-1, and Fra-2) and c-Jun family members (c-Jun, JunB, and JunD) through qPCR. Results demonstrate a significant increase in expression levels for Fos family members c-Fos, Fra-1, and FosB and a significant repression in Fra-2 (Figure 6A). There was no change in the Jun family members c-Jun and JunB; however; JunD was significantly repressed (Figure 6B). The increase in c-Fos and Fra-1 expression also correlated with an increase in c-Fos and Fra-1 phosphorylation observed through western blot (Figure 6C). To determine if the observed increase in AP-1 gene expression and phosphorylation correlated with an increase in target gene transcription, the MAPK and AP-1 mediated genes, SerpinE1, Nme1, Plaur, Zeb1, Snail, and E-cadherin were analyzed by qPCR. Results demonstrate a significant increase in AP-1 regulated genes SerpinE1, Plaur, Zeb1, and Snail (Figure 6D).

## DISCUSSION

The global effects of a miRNA can be determined by multiple factors including: the heterogeneity of the miRNA seed site, the expressed mRNA isoforms, and alterations to the mRNA 3′UTR. Here we have explored the overall effect of miR-155 expression in the MCF-7 breast cancer cell line through evaluation of the cellular transcriptome using next generation sequencing. We demonstrate that miR-155 induces increased MAPK signaling evident through both increased protein phosphorylation of MAPK signaling components (Fos, Fra-1, ERK1/2) and gene expression (Fos, Fra-1, p38). Additionally, miR-155 induced alterations in MAPK signaling genes through loss of 3′UTRs and isoform variances. Shortening of 3′UTR will result from multiple different factors such as 3′UTR methylation and alternative cleavage and polyadenylation (APA) sites giving rise to gene isoforms with truncated 3′UTRs [45-47]. Breast cancer cell lines demonstrated increased expression of 3′UTR shortening due to APA sites, which is associated with increased cellular proliferation [45, 46]. In accordance with this, our data demonstrated loss of miR-155 targeting through shortening of mRNA 3′UTRs at the site of 8-mer seed sites and increased tumorigenesis *in vivo*. miR-155 is known to target genes such as SOCS1 and TP53INP1 enhancing proliferation in breast cancer cell lines [2, 48]. One intriguing idea is that the observed loss of 3′UTR and subsequent enhanced expression of MAPK associated genes is a result of miR-155 induced proliferation initiated by suppression of miR-155 targets such as SOCS1 and TP53INP1. This would result in a feed forward mechanism which propagates proliferation and tumorigenesis; however, this still remains to be fully evaluated. Evaluation of induced 3′UTR shortening is emerging as a novel layer to miRNA targeting and demonstrates how miRNA/mRNA targeting can be cell type specific. Additionally, loss of miRNA targeting distinguishes a method for how changes in the transcriptomes can mediate the miRNA induced phenotypic profile of a cell. Loss of 3′UTR in the MAP3K10 gene are demonstrated here and previously [44]. Conversely 3′UTR shortening was not observed in the human monocytic cell line THP-1, and miR-155 was able to target the mRNA transcript in this cell line [49]. Interestingly, successful miR-155 targeting of MAP3K10 resulted in a global repression of MAPK signaling. This is reciprocal to what we show where, which is loss of miR-155 targeting of MAP3K10, increased MAP3K10 expression, and enhanced MAPK signaling. MAP3K10 alone is not the only driving factor in enhancing MAPK signaling, as we demonstrate enhanced expression of multiple MAPK signaling components. Like MAP3K10, Rac1, MAP3K14, and CEBPβ all demonstrated loss of miR-155 target site through 3′UTR shortening. In addition isoform variance was observed for the MAPK effector, RSK2. It is worth noting that the differences in mRNA 3′UTR architecture and isoform expression can influence the effects of a miRNA just as much as the miRNA itself. The inability of miRNAs to function similarly in different cellular systems can be speculated to be a result of variances in isoform transcripts and 3′UTR, drawing light on these differences aid in enhancing our knowledge of miRNA.

## MATERIALS AND METHODS

### Cells and Reagents

The MCF-7 human breast cancer cell line was acquired from American Type Culture Collection (Manassas, VA). Liquid nitrogen stocks were made upon receipt and maintained until the start of study. ERE–luciferase and/or qPCR for ER and PgR were used to confirm sustained estrogen responsiveness of ER^+^ cell line. Morphology and doubling times were also recorded regularly to ensure maintenance of phenotype. Cells were used for no more than 6 months after being thawed. Cells were maintained in Dulbecco's modified Eagle's medium (DMEM; pH 7.4; Invitrogen Corp., Carlsbad, CA) supplemented with 10% fetal bovine serum (Hyclone, Salt Lake City, UT), 1% non-essential amino acids, minimal essential amino acids, sodium pyruvate, penicillin/streptomycin (pen/strep), and insulin under mycoplasma-free conditions at 37°C in humidified 5% CO2 and 95% air.

### Generation of miR-155 cell line

miR-155 plasmid construct was constructed as previously described [50]. Parental MCF-7 cells were transfected with pmscv-pre-mir-155 or pmscv-vector plasmid using Lipofectamine 2000 at 1ug/ul OPTI-MEM (Invitrogen, Grand Isles, NY) as per manufacturer's protocol. Cells were grown in a 100mm dish. In a 5ml tube, 5ug pre-mir-155 or vector plasmid was added to 100ul serum free opti-MEM then 15ul Lipofectamine was added. Following 30 minutes opti-MEM containing plasmid was added to MCF-7 cells. The following day cells were treated with 300 ng/ml puromycin. Cells were maintained in 10% DMEM and treated with 300 ng/ml puromycin every two days for 2 weeks. Colonies were pooled and verification of mature miR-155 overexpression was confirmed using qPCR for mature miR-155.

### RNA Extraction and Quantitative Real Time RT-PCR

MCF-7-pmscv-vector and MCF-7-miR-155 cells were harvested and total RNA extracted using Qiagen RNeasy purification system per manufacturer's protocol (Qiagen, Valencia, CA). Quantity and quality of the RNA were determined by absorbance at 260 and 280 nm using the NanoDrop ND-1000. 1ug of total RNA was reverse-transcribed using the iScript kit (BioRad Laboratories, Hercules, CA) and qPCR was performed using SYBR-green (Bio-Rad Laboratories, Hercules, CA). Genes amplified, n = 4 independent biological replicates.

### Crystal Violet Assay

MCF-7-pmscv-vector and MCF-7-miR-155 cells were grown in 10% DMEM for 24 hours and then seeded on 48 well plates (7,000 cells per well) for 24 hours prior to a one time treatment with PD184352 (5μM) (Tocris, Bristol, United Kingdom), RWJ 67657 (10μM) (Johnson and Johnson Pharmaceutical Research & Development, L.L.C., Raritan, NJ), SP600125 (20μM) (Tocris, Bristol, United Kingdom), BIRB (0.5μM) (Selleckchem, Houston, TX)or DMSO. After 72 hours, cells were washed once with PBS and fixed and stained using 0.1% Crystal Violet (in 20% methanol) for 10 minutes. Cells were washed 1X with water and then lysed with 1% SDS. A Gene5 plate reader was used to read absorbance at wavelength 570. Each cell line was normalized to its respective DMSO treated group, n=3.

### Western Blot Analysis

MCF-7-pmscv-vector and –miR-155 cells were grown in 10% FBS DMEM. Cells were washed with PBS and lysed with M-Per lysis buffer supplemented with 1% protease inhibitor and 1% phosphatase inhibitors (cocktail I/II) (Invitrogen, Grand Isles, NY). Supernatant containing protein extracts was obtained through centrifugation at 12,000 RPM for ten minutes at 4°C. Protein extracted per sample was determined by absorbance at 260 and 280 nm using the NanoDrop ND-1000. Proteins were heat denatured and 100μg of protein were loaded per lane on Bis-Tris-nuPAGE gel (Invitrogen, Grand Isles NY). Protein was transferred to nitrocellulose through iBlot and iBlot transfer stacks as per manufacturer's protocol (Invitrogen, Grand Isles, NY). Nonspecific binding of primary antibody was blocked by incubation in 3% milk in 1% TBS-T for 1 hour. Following blocking, membrane was incubated overnight with primary antibody for p-c-Fos, p-Fra-1, and total Fra-1 (diluted 1:1000) (Cell Signaling Technology, Beverly MA). The following day membrane was washed and followed by three fifteen minute washes in 1% TBS-T. Membrane was incubated for 1 hour in secondary antibody 1:10,000 dilution (LiCor Bioscience, Lincoln NE) followed by three ten minute washes in 1% TBS-T. Band density was determined by Odyssey gel imager. Loading control protein was Rho GDI-α (Santa Cruz Biotechnology, Santa Cruz, CA) diluted 1:500. Experiments were conducted n=3 biological replicates with representative images shown.

### Proteome Profiler Human Phospho-Kinase Array

MCF-7-pmscv-vector and MCF-7-miR-155 cells were seeded in 10 cm2 plates 2 ×10^6^ and allowed to attach overnight. The next day cells were lysed according to the manufacturer's instructions (R&D Systems Cat# ARY003B). A single blot was used for each cell line and processed as per manufacturer's instructions with the exception being that the secondary antibody used for blots was infrared 1:10,000 dilution (LiCor Bioscience, Lincoln NE). Imaging was performed on Odyssey LiCor gel imager. Individual dots were quantified using the Odyssey software and normalized using the average of positive controls as indicated on the blot.

### Xenograft Studies

4-6 wks. old ovariectomized SCID/CB17 female mice (Charles River Laboratories; Wilmington, MA) were allowed a period of adaptation in a sterile and pathogen-free environment with food and water ad libitum. Cells were harvested in the exponential growth phase using a PBS/EDTA solution and washed. Viable cells (5 × 10^6^) in 50 μl of sterile PBS suspension were mixed with 100 μl Reduced Growth Factor Matrigel (BD Biosciences, Bedford, MA). Injections were administered into the mammary fat pad using 27 ½ gauge sterile syringes. Animals were divided into treatment groups of five mice each: MCF-7-pmscv-vector and MCF-7 cells transduced to overexpress miR-155. All procedures in animals were carried out under anesthesia using a mix of isofluorane and oxygen. Tumor size was measured every 2-3 days using digital calipers. The volume of the tumor was calculated using the formula: 4/3π LS2 (L= larger radius; S = shorter radius). At necropsy, animals were euthanized by cervical dislocation after exposure to CO^2^. Tumors were removed and frozen in liquid nitrogen or fixed in 10% formalin for further analysis. All procedures involving these animals were conducted in compliance with State and Federal laws, standards of the U.S. Department of Health and Human Services, and guidelines established by Tulane University Animal Care and Use Committee. The facilities and laboratory animals program of Tulane University are accredited by the Association for the Assessment and Accreditation of Laboratory Animal Care.

### Next Generation Sequencing

Full methods and parameters of alignment were performed as previously described [44]. Briefly, MCF-7-pmscv-vector and –miR-155 cell lines were harvested for total RNA extraction using the mRNeasy (Qiagen) kit. Next Generation Sequencing (NGS) was performed using the Illumina Genome Analyzer II. Results were aligned in the fastq format against the annotated human reference genome (Hg19). Reads per kilobase of exon model per million mapped reads (RPKM) were generated through SAMMate which compiled alignments generated with the Novoalign (http://www.novocraft.com) and TopHat aligners. Use of TopHat allowed for mapping of exon-exon junctions that may have initially read as unmapped reads [51]. Fold change in gene expression was calculated using RPKM. Gene expression analysis was viewed in Integrative Genomics Viewer (IGV).

### Statistical Analysis

Statistical Analysis was performed using Graph Pad Prism 5. Student's t test was used to determine p values and statistically significant values had a p-values of <0.05.
